# Complement profiling of sural nerves in chronic-inflammatory demyelinating polyneuropathy

**DOI:** 10.1007/s00401-025-02936-w

**Published:** 2025-09-19

**Authors:** Frauke Stascheit, Andreas Roos, Christina B. Schroeter, Johanna Katrin Thomas, Katrin Hahn, Hannah Preßler, Andreas Hentschel, Beate Schlotter-Weigel, Benedikt Schoser, Tobias Ruck, Andreas Meisel, Werner Stenzel, Corinna Preusse

**Affiliations:** 1 Charité—Universitätsmedizin Berlin, Department of Neurology With Experimental Neurology, Corporate Member of Freie Universität Berlin and Humboldt Universität Zu Berlin, Charitéplatz 1, 10117 Berlin, Germany; 2 Charité—Universitätsmedizin, Corporate Member of Freie Universität Berlin, Humboldt-Universität Zu Berlin, Neuroscience Clinical Research Center, Berlin, Germany; 3 Department of Neurology, Medical Faculty and University Hospital Düsseldorf, Heinrich Heine University Düsseldorf, Moorenstr. 5, 40225 Duesseldorf, Germany; 4 Department of Pediatric Neurology, Centre for Neuromuscular Disorders, University Duisburg-Essen, 45147 Essen, Germany; 5 Brain and Mind Research Institute, Children’s Hospital of Eastern Ontario Research Institute, Ottawa, ON K1H 8L1 Canada; 6 Charité—Universitätsmedizin Berlin, Department of Physical Medicine, Corporate Member of Freie Universität Berlin and Humboldt-Universität Zu Berlin, Charitéplatz 1, 10117 Berlin, Germany; 7 Charité—Universitätsmedizin Berlin, Department of Neuropathology, Corporate Member of Freie Universität Berlin and Humboldt-Universität Zu Berlin, Charitéplatz 1, 10117 Berlin, Germany; 8 Charité—Universitätsmedizin Berlin, Department of Neuropaediatrics, Corporate Member of Freie Universität Berlin and Humboldt-Universität Zu Berlin, Augustenburger Platz 1, 13353 Berlin, Germany; 9 Center for Stroke Research Berlin, Charité—Universitätsmedizin Berlin, Corporate Member of Freie Universität Berlin, Humboldt-Universität Zu Berlin, Berlin, Germany; 10 Leibniz-Institut Für Analytische Wissenschaften—ISAS—E.V., 44139 Dortmund, Germany; 11 Friedrich-Baur-Institut an der Neurologischen Klinik Und Poliklinik, LMU Munich, Munich, Germany; 12 Department of Neurology, Ruhr University Bochum, BG University Hospital Bergmannsheil, Bochum, Germany; 13 BG University Hospital Bergmannsheil, Heimer Institute for Muscle Research, Bochum, Germany

**Keywords:** Chronic-inflammatory demyelinating polyneuropathy, Complement profiles, Sural nerve, Gene expression, Proteomics

## Abstract

Chronic-inflammatory demyelinating polyneuropathy (CIDP) is a rare immune-mediated polyneuropathy causing substantial disability. While both cell-mediated and humoral mechanisms contribute to CIDP, the role of complement remains poorly understood. Considering the rise of complement-targeted treatment, it is crucial to examine the role of complement in CIDP. In this cross-sectional, study, sural nerve biopsies from 55 CIDP patients were analyzed using histopathology, gene- and protein-based techniques, comparing them to two non-diseased controls (NDCs), as well as 8 patients with hereditary neuropathy (HN) and idiopathic axonal neuropathy (IPN). Overall, 94% (n = 52) revealed abnormal and prominent deposition of terminal complement complex C5b-9 on endoneurial capillaries. Patients with significant complement deposition presented with a progressive disease course (n = 52) and the number and distribution of infiltrating CD8 + T cells and CD68 + macrophages, since a basic immunological paradigm holds that those two may form an immunological synapse, correlated with clinical disease severity as measured by inflammatory neuropathy cause and treatment sensory sum (INCAT) score (*p* < 0.001). Furthermore, changes in abundances of complement proteins as unveiled by untargeted proteomics accord with changes on transcript level as identified by targeted gene expression studies. In contrast, there was no complement deposition in NDC nor DC. This study provides an extensive evaluation of sural nerve specimens of CIDP patients finding a marked involvement of complement supporting the postulated concept of complement mediated demyelination in CIDP. Our results support the approach of targeting the complement system as a new and promising therapeutic strategy—at least in a subgroup of CIDP. Further research is warranted to unravel the functional implications and role of complement in CIDP progression and optimize patient care. *Clinical Trial Registration*: The study is registered under the German clinical trial registry (https://www.drks.de), DRKS0003245.

**Supplementary Information:**

The online version contains supplementary material available at 10.1007/s00401-025-02936-w.

## Introduction

Chronic-inflammatory demyelinating polyneuropathy (CIDP) is an autoimmune disease affecting the peripheral nervous system. This most frequent autoimmune polyneuropathy is characterized by the predominant demyelination of motor and sensory nerves [11, 26]. CIDP follows a relapsing–remitting or progressive disease course and is classified according to its clinical manifestation into “typical” CIDP, characterized by proximal and distal symmetric and predominant weakness, and “atypical” CIDP, currently redefined as CIDP variants [44].

Current treatment strategies for CIDP include corticosteroids, intravenous (IVIg) or subcutaneous immunoglobulins, and plasma exchange, which are effective in the majority of patients [43]. Recently, the neonatal Fc receptor (FcRn) inhibitor efgartigimod has demonstrated clinically proven efficacy in a phase III trial and has been approved for the treatment of CIDP [3], thereby expanding the range of available therapeutic options. However, the response to these treatments remains heterogeneous and often requires long-term therapy leading to substantial burden of disease and disability in a subgroup of patients over time [37].

Differences in clinical presentation and therapeutic response are probably due to different pathophysiological mechanisms but thus far remain poorly understood [2, 41]. In addition to autoreactive T cells [22, 24, 40], auto-antibodies (Abs) directed against ganglioside Abs [23] and paranodal structures [9] are thought to play a crucial role in the etiology. However, these antibodies are detectable only in a small subset of patients and, according to the current EAN/PNS guidelines [44], define distinct autoimmune nodopathies characterized by therapy-refractory disease courses and pathognomonic clinical features. In the vast majority of CIDP cases, a specific target antigen remains unidentified. Although the primary mechanism of efgartigimod is the depletion of circulating IgG via FcRn blockade, its clinical efficacy in CIDP—where no specific target antibody has been defined—suggests that indirect downstream effects, such as reduced immune complex-mediated complement activation or modulation of proinflammatory cytokine pathways, may contribute to its therapeutic action. The rapid clinical response to both plasmapheresis and efgartigimod [3] supports the involvement of a circulating factor, potentially including noncompact myelin-specific antibodies, cytokines, or components of the complement cascade [11, 26]. Nevertheless, direct evidence for complement involvement in CIDP remains scarce.

The complement system is a complex cascade that modulates tissue homeostasis and contributes to immune surveillance by interacting with the innate and adaptive immune systems [14]. If disrupted, it is crucial for triggering several autoimmune neurological disorders [10, 14]. Complement activation can result from the classical (via C1q), lectin, and alternative (via C3) pathways. Subsequently, the effector proteins C3a, C3b, and C5a and the membrane attack complex (C5b-9) are generated leading to target cell lysis [14].

In earlier studies, sural nerve specimens from patients with CIDP showed deposition of complement factor C3 on the myelin sheath [15, 19]. However, these studies included only few patients (n = 4 resp. n = 7). Moreover, complement inhibition therapy has improved muscle weakness in an experimental rat model of CIDP [17]. Apart from complement deposition on the myelin sheath, increased serum and cerebrospinal fluid (CSF) levels of C5a and the soluble terminal complement complex C5b-9 have also been reported in newly diagnosed, treatment-naïve CIDP patients compared to controls [36]. Moreover, recently presented results from the C1s-inhibitor riliprubart, show a clinically meaningful improvement in clinical disability scores and support the crucial involvement of complement in the pathogenesis of CIDP [39].

Considering the current emergence and success of complement-targeted therapies [20, 29], it is, therefore, crucial to examine the role of the complement system in CIDP to potentially tackle the unmet needs of more targeted therapeutic strategies and improve patient care.

Hence, in this study, we examine complement profiles on sural nerve specimens in a large cohort of CIDP patients compared to non-diseased controls (NDC) and diseased controls (DC) with non-inflammatory neuropathies using immunohistochemistry, quantitative PCR (qPCR), and proteomic analysis in an explorative manner to further unravel its pathophysiological role in CIDP.

## Materials and methods

### Patient recruitment and clinical data

Patients who received a sural nerve biopsy were recruited from January 2006 to May 2023 at the CIDP outpatient Department and Department of Neuropathology at the Charité-Universitätsmedizin Berlin, Germany. Study inclusion required patients above the age of 18 years, independent of disease duration and severity, to meet the national and European guidelines for diagnosis of CIDP according to EAN/PNS criteria [44]. In accordance with these criteria, patients with confirmed paranodal auto-antibodies (anti-NF155, anti-CNTN1, and anti-Caspr1) were excluded, as such cases are classified as autoimmune nodopathies rather than CIDP. Due to diagnostic doubts or refractory status, patients received a sural nerve biopsy. To minimize treatment-related effects on complement deposition, we excluded patients who had received corticosteroids or intravenous immunoglobulin (IVIG) within 6 weeks prior to sural nerve biopsy. This interval was chosen based on published recommendations for the assessment of complement activity in serum and plasma [1], as it allows for normalization of transient complement suppression following these treatments. The treatment-free interval was verified through detailed review of medical records.

DC served a total of 8 sural nerves with a morphological diagnosis of chronic mildly active axonal damage without overt signs of any inflammatory alterations. These patients were classified as idiopathic neuropathy (IPN) as an extended diagnostic approach according to the EAN/PNS guidelines, which did not reveal any underlying cause for the neuropathy. In addition, one sural nerve biopsy with clinical manifestation of hereditary neuropathy (HNP) and a genetic test confirming the diagnosis was also examined for complement deposition. Moreover, we included two non-disease control (NDC), which received a sural nerve biopsy to exclude potential somatic cause of neuropathic symptoms. One NDC was recruited from the Department of Neurology, Friedrich-Baur Institute, LMU Munich, Germany.

Patients were examined by a team of peripheral nerve specialists. Socio-demographics and current medication were documented. The medical research council sum scale (MRC-SS) was assessed to measure muscle strength at the impairment level [25]. For the MRC-SS the following muscles were tested on both sides: shoulder abduction, elbow flexion, wrist extension, index finger extension, hip flexion, knee extension, ankle dorsiflexion, and extension of the big toe. A sum score of 80 indicates normal muscle strength. The adjusted inflammatory neuropathy cause and treatment disability score (INCAT-DS) was used to assess the clinical disability in daily arm and leg mobility, which has evolved as the most established primary outcome in clinical trials [21, 30]. In addition, we assessed CSF parameters, comorbidities, treatment regime (corticosteroids, IVIg, plasmapheresis, and immunosuppressive therapies) and treatment response and further categorized the patients according to their CIDP disease activity status (CDAS) into active disease status with relapsing or chronic progressive disease course or remission [18].

### Sural nerve specimens

After shock cryofixation and diagnostic processing all sural nerve specimens had been cryopreserved at − 80 °C before analysis according to the predefined standard operating procedure of the Department of Neuropathology at the Charité-Universitätsmedizin, Berlin, Germany [34, 35]. For mass spectrometry-based analysis, sural nerve samples were transferred on dry ice to the Leibniz-Institute for Analytical Sciences–ISAS–e.V. and stored at − 80 °C prior to sample processing. Sural nerve biopsies without any evidence of pathological alterations by histology, semithin studies, fiber teasing, and ultrastructural analysis were defined as non-diseased control.

### Histological and immunohistochemical analysis

Routine stains, immunohistochemical-, and double-immunofluorescence reactions were performed as previously described [34, 35]. The following antibodies were used for staining procedures: C5b-9 (Dako, aE11, 1:200), CD8 (Dako, C8/144B, 1:100), CD45 (Dako, 2B11, 1:400), CD68 (Dako, EBM11, 1:100). The presence of C5b-9 was further visually quantified in high, medium and low staining intensity (examples given in eFigure 1). CD8 + T cells were graded for none (0–4 cells/10 HPF), few (5–12 cells/10 HPF) or multiple (> 12 cells/10 HPF). HPFs, based on the microscope used and the respective oculars (Olympus WH10x-H/22) ≙ 0.16 mm^2^. CD68 + macrophages were graded semi-quantitatively for few and diffusely distributed (endoneurium); grade 1, multiple and increased but diffusely distributed; grade 2, or many and clustering with T cell accumulation; grade 3. HPFs, based on the microscope used and the respective oculars (Olympus WH10x-H/22) ≙ 0.16 mm^2^. Antibodies were detected with the immunoperoxidase method. All staining procedures were performed in the same laboratory on a Benchmark XT immunostainer (Ventana, Tucson, AZ) [34].

### Real-time qPCR

RNA extraction from the nerve tissue samples, reverse transcription, and quantitative PCR reactions were performed as previously described [35]. In short, total RNA was extracted from sural nerve specimens, and cDNA was synthesized using the High-Capacity cDNA Archive kit (Applied Biosystems, Foster City, CA). For qPCR reactions, 10 ng of cDNA was used. For subsequent analysis, the QuantStudio 6 Flex System (Applied Biosystems) was used with the following running conditions: 95 °C 0:20, 95 °C 0:01, and 60 °C 0:20, 45 cycles (values above 40 cycles were defined as not expressed). All targeted transcripts were run as triplicates. The TaqMan® Gene Exp Assay (Life Technologies/ThermoFisher) are as follows: *APRIL/TNFS13* Hs00182565_m1, *BAFF/TNFSF13B* Hs00198106_m1, *C1QA* Hs00706358_s1, *C3* Hs00163811_m1, *C4A* Hs00246758_m1, *C5* Hs01004342_m1, *C6* Hs01110040_m1, *C8A* Hs00175098_m1, *C9* Hs01036216_g1, *TNFA* Hs00174128_m1.

*PGK1* Hs99999906_m1 was included as an internal control to normalize the relative expression of the targeted transcripts. Gene expression was illustrated by the fold-change values compared with that in NDCs.

### Lysate generation and processing for proteomic deep mapping

The entire nerve sample was lysed in 200 µl of 50 mM Tris–HCl buffer (pH 7.8), containing 5% SDS and cOmplete ULTRA protease inhibitor (Roche), using the Bioruptor® (Diagenode) for 10 min with 30-s on/off cycles for a total of 10 cycles at 4 °C. An additional sonication step was performed with an ultrasonic probe (30 s, alternating 1 s on and 1 s off, amplitude 40%) to ensure thorough lysis. This was followed by centrifugation at 20,000 g for 15 min at 4 °C. The protein concentration in the resulting supernatant was measured using a BCA assay following the manufacturer's instructions. Disulfide bonds were reduced by adding 10 mM TCEP at 37 °C for 30 min, and free sulfhydryl groups were alkylated using 15 mM IAA at room temperature, in the dark, for 30 min. For proteolysis, 100 µg of protein from each sample was processed using the S-Trap protocol (Protifi) with a 20:1 protein-to-trypsin ratio. The trypsin digestion step was carried out for 2 h at 42 °C and stopped by acidifying the sample with formic acid to achieve a pH below 3.0. Samples were dried using a Speedvac (Thermo Fisher Scientific, Waltham, MA, USA) and dissolved in 0.1% TFA to achieve a 0.5 µg/µl concentration.

All hydrolyzed samples were checked for completeness of digestion after desalting through monolithic column separation (PepSwift monolithic PS-DVB PL-CAP200-PM, Dionex) on an Ultimate 3000 HPLC system (Dionex, Germering, Germany) via direct injection of 0.5 μg of sample. A binary gradient (solvent A: 0.1% TFA, solvent B: 0.08% TFA, 84% ACN) was applied, transitioning from 5% to 12% B over 5 min, followed by 12–50% B over 15 min, at a flow rate of 2.2 μL/min and 60 °C. UV detection was performed at 214 nm (doi.org/10.1016/j.jprot.2011.11.016).

### Mass spectrometry-based proteomic profiling

All samples were analyzed using an UltiMate 3000 RSLC nano-UHPLC system coupled to a QExactive HF mass spectrometer, with 1 µg of peptide used for each analysis. Initially, the samples were transferred to a 75 µm × 2 cm, 100 Å, C18 pre-column at a 10 µl/min flow rate for 20 min. Separation was then performed on a 75 µm × 50 cm, 100 Å, C18 main column at a 250 nl/min flow rate. The separation utilized a linear gradient composed of solution A (99.9% water, 0.1% formic acid) and solution B (84% acetonitrile, 15.9% water, 0.1% formic acid), with a pure gradient length of 120 min, transitioning from 3% to 45% solution B. The gradient profile was as follows: 3% solution B for the first 20 min, 3–35% over 120 min, followed by three washing steps at 95% solution B, each lasting 3 min. After the final wash, the system was equilibrated for 20 min. For MS survey scans, the following settings were used: MS was operated in data-dependent acquisition mode (DDA) with full MS scans from 300 to 1600 m/z (resolution 60,000) with the polysiloxane ion at 371.10124 m/z as lock mass. Maximum injection time was set to 120 ms. The automatic gain control (AGC) was set to 1E6. For fragmentation, the 15 most intense ions (above the threshold ion count of 5E3) were chosen at a normalized collision energy (nCE) of 27% in each cycle, following each survey scan. Fragment ions were acquired (resolution 15,000) with an AGC of 5E4 and a maximum injection time of 50 ms. Dynamic exclusion was set to 15 s.

### Bioinformatic analysis of protein data

Z-scoring was performed with the raw abundance values obtained and normalized by the Proteome Discoverer 2.5 software (Thermo Scientific) to create and visualize the heatmap. Subsequently, the received data were filtered for complement proteins. Only proteins that could be identified with at least two unique peptides were considered reliable identifications and retained. After filtering the data, the visualization was displayed in a heatmap using orange data mining software. Clustering was performed based on similarity with hierarchical clustering on Euclidean distances and with average linkage of the individual samples. The mass spectrometry proteomics data have been deposited to the ProteomeXchange Consortium via the PRIDE partner repository with the data set identifier PXD056286 [33].

### Statistical analysis

Descriptive statistics are reported as means and standard deviations (SDs), medians and interquartile ranges (IQRs) for continuous variables, and absolute and relative frequencies for nominal data. All statistical analyses were performed using GraphPad Prism V10.2.2.

Mann–Whitney tests were performed to compare levels of complement components between patients and controls. Their correlations with clinical disease parameters were analyzed using Spearman's rank correlation coefficient. A two-tailed *p* value < 0.05 was considered statistically significant.

## Results

### Demographics and characteristics of CIDP patients

In this study, we included 55 patients with CIDP, of which a typical disease course was observed in 36 (65%) patients (Table 1). The median age at manifestation of symptoms was 62.0 years (IQR 50.0–69.0) and at diagnosis 65.0 years (IQR 55.0–69.0), with no relevant difference between the group of patients with typical course and CIDP variant. The majority of patients were of male sex (n = 35, 64%). The time between first manifestation and diagnosis was quite comparable between the two groups, with 34.7 months (SD 38.9) in typical CIDP and 38.9 (SD 41.6) months in patients with a CIDP variant, which may be the reason for the slightly higher disability in this subgroup at time of sural nerve biopsy (median inflammatory neuropathy cause and treatment sensory sum score (INCAT) score in typical CIDP 0 (IQR 0.0–2.0) for arm, 1.0 (IQR 1.0–2.0) for leg; median INCAT score in CIDP variant 1 (IQR 0.0–2.0) for arm, 1.0 (IQR 1.0–1.0) for leg. Regarding diagnostic criteria, all patients had albumin cytological dissociation in CSF with a mean protein level of 678.5 mg/dl (SD 346.4), without relevant difference between the groups. One-third of patients with typical CIDP (n = 19, 35%) and 19% (n = 6) in the CIDP variant had purely demyelinating neurography at diagnosis. There was a therapeutic delay in the CIDP variant group with 10.2 months (SD 11.6) compared to 5.7 months (SD 7.6) in the typical CIDP group. The majority of patients presented with a progressive disease course (n = 40, 73%), with a higher percentage in the CIDP variant group (84%, n = 16 vs. 66%, n = 24 in typical CIDP). All patients had a treatment history with IVIg and corticosteroids and were treatment refractory, which was why a sural nerve biopsy was performed. The mean time from the last treatment (steroids or IVIG) until nerve biopsy was 7.9 weeks (SD 1.1).

**Table 1:** Demographical and clinical characteristics of CIDP patients and controls

	Total CIDP	Typical CIDP*	CIDP variant	NDC	HNP	IPN
N (%)	55	36 (65%)	18 (13%)	2	1	7
*Sex*						
Male, N (%)	35 (64%)	22 (61%)	13 (68%)	0	0	5 (71%)
Age at manifestation, median (IQR)	62.0 (50.0–69.0)	63.5 (55.3–69.8)	61.0 (50.5–69.0)	35.2	41	57.0 (45.3–71.0)
Age at diagnosis, median (IQR)	65.0 (55.0–73.0)	66.5 (59.3–74.5)	64.0 (55.5–73.0)	35.8	42	58.0 (46.0–72.0)
Time between manifestation AND diagnosis (months), mean (SD)	34.7 (38.9)	33.9 (40.0)	38.0 (41.6)	**–**	29.0 (22.1)	32.8 (35.1)
*Clinical presentation*						
Sensory	55 (100%)	33 (92%)	19 (100%)	**–**	1 (100%)	7 (100%)
Weakness		32 (89%)	18 (95%)		1 (100%)	7 (100)
Tremor	50 (91%)	2 (6%)	0 (0%)		**–**	**–**
Neuropathic pain	2 (4%)	12 (33%)	10 (52%)		**–**	**–**
Ataxia	27 (49%)	33 (92%)	19 (100%)		**–**	**–**
Cranial nerve	51 (93%)	1 (3%)	0 (0%)		**–**	**–**
Involvement	1 (2%)					
Cerbrospinal fluid						
Cell number (MEAN ± SD) (CELL/µL)	2.1 (1.5)	2.1 (1.3)	2.0 (2.2)	**–**	**–**	2 (1.2)
Protein level (MEAN ± SD) (MG/DL)	678.5 (346.4)	689.7 (335.4)	708.7 (443.9)	**–**	**–**	384 (112)
(Missing)	(8)					
*Neurography, N (%)*						
Demyelinating	19 (35%)	13 (36%)	6 (19%)	**–**	**–**	**–**
Pure axonal	0 (0%)	0 (0%)	0 (0%)	**–**	**–**	**–**
Axonal-Demyelinating	30 (55%)	18 (50%)	12 (63%)	**–**	1 (100%)	7 (100%)
(Missing)	(6)					
*Incat score at diagnosis (IQR)*						
ARM	1 (0–2.0)	0 (0–2.0)	1 (0–2.0)	**–**	**–**	**–**
LEG	1 (1.0–2.0)	1 (1.0–2.0)	1 (1.0–1.0)			
(Missing)	(13)					
MRC-sum score at diagnosis (IQR)	76.0 (70.0–80.0)	75.0 (70.0–80.0)	78.0 (76.0–78.0)	**–**	**–**	**–**
(Missing)	(4)					
Clinical disease course, N (%)				**-**	**-**	**-**
Progressive	40 (73%)	24 (66%)	16 (84%)			
Relapsing	7 (13%)	5 (14%)	2 (11%)			
Stable	4 (7%)	3 (8%)	1 (5%)			
(Missing)	(4)					
*CIDP-specific treatment in overall disease course, N (%)*						
Corticosteroids	23 (42%)	14 (39%)	9 (47%)	**–**	**–**	**–**
Intravenous immunoglubilines	37 (67%)	22 (61%)	13 (68%)			
Combination therapy with Azathioprine	6 (11%)	3 (8%)	2 (11%)	**–**	**–**	**–**
Other (Plasmapheresis, Rituximab)	6 (11%)	3 (8%)	5 (26%)	**–**	**–**	**–**
(Missing)	(8)					
Time to first treatment in months, mean (SD)	7.9 (11.8)	5.7 (7.6)	10.2 (11.6)	**–**	**–**	**–**

Data are mean (SD) or median (IQR) and n (%) for the baseline variables

*CIDP*—chronic-inflammatory demyelinating polyneuropathy, *HNP* hereditary neuropathy, *IPN* idiopathic neuropathy, *INCAT* inflammatory neuropathy cause and treatment sensory sum score, *IQR* interquartile range, *MRC* medical research council sum score, *NDC* non-diseased controls. *SD* standard deviation, – not applicable

*According to EAN/PNS Guidelines from 2021

### Immunohistochemical characteristics and gene expression levels of CIDP patients’ sural nerve biopsies

**Fig. 1:**
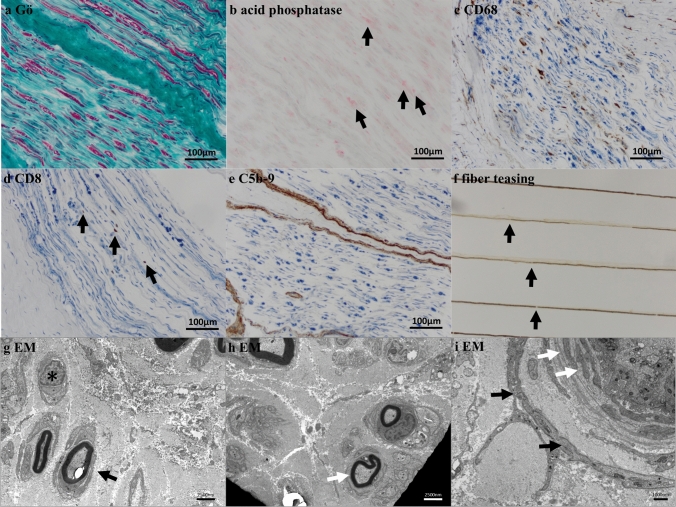
Morphology of a representative sural nerve specimen in CIDP. **a** Gömöri trichrome staining of sural nerve of CIDP showing loss of myelinated axons. **b** Acid phosphatase activity demonstrates activated macrophages, as also seen in **c**, with endoneurial clusters of CD68 + macrophages detectable in all (n = 55) of sural nerve specimens with variable densities. **d** Endoneurial clusters of CD8 + T cells are detectable in all samples (n = 55), as are **e** terminal complement deposits (C5b-9) on endoneurial capillaries. These deposits are detectable in 95% (n = 52) of sural nerve specimens—again with variable intensity. **f** Fiber teasing shows segmental hypo-/demyelination and shortening of segments > 60% of normal length (black arrows). Electron microscopy **g**, **h** shows hypomyelination (black arrow) and loss of unmyelinated axons (*), as well as onion bulb formation (white arrow). Electron microscopy of a capillary (white asterisk) in a sural nerve fascicle **i** demonstrating multiple basement membrane duplications (white arrows), with fibroblasts surrounding the basement membrane duplications indicating sustained and repetitive capillary damage (black arrows)

All sural nerve specimens were analyzed immunohistochemically and stained for C5b-9, CD8^+^ T cells, myelin loss (by trichrome staining) and CD68^+^ macrophages (see Fig. 1). Most CIDP patients presented with a marked endoneurial capillary staining of C5b-9 (n = 52/55, 95%; Fig. 1e**, **Table 2**)**. We did not observe complement deposition on Schwann cells. Interestingly, those three patients with only minor complement deposition all showed clinical sensory symptoms and neuropathic pain and did not present with a progressive disease course. There was no significant difference in amounts of complement deposition when stratified by disease subtypes (classical CIDP, CIDP variant; *p* = 0.704, Mann–Whitney test). Moreover, in almost all patients endoneurial CD8^+^ T cells clustering with groups of CD68^+^ macrophages in the endoneurium in addition to marked myelin loss and active phagocytosis as illustrated by acid phosphatase-positive endoneurial monocytes could be observed (see Figs. 1a–d**, **). Co-staining of macrophage markers (CD11b, CD68 or Siglec1/CD169) and complement deposition (C5b-9) also clearly visualize proximity of macrophages and complement positive capillaries (eFigure 2). Furthermore, semithin sections, fiber teasing, and electron microscopy showed hypomyelination, loss of myelinated and unmyelinated axons, and onion bulb formation (see Fig. 1g, h). Exemplary staining of healthy nerve is shown in eFigure 3. Enlargement of capillary walls due to basement membrane duplications within sural nerve fascicles demonstrates capillary degeneration and regeneration (see Fig. 1i). The DC group of patients with IPN and HNP showed either no or faintly detectable little (+; see semiquantitative scale) C5b-9 deposits on endoneurial capillaries. An example of such a biopsy is in eFigures 4 and 5, respectively.

**Table 2:** Histological findings of sural nerve biopsies

	Total CIDP	Typical CIDP	CIDP variant
(%)	55	36 (65%)	18 (13%)
*Complement deposition, N (%)*			
High	7 (13%)	4 (11%)	3 (16%)
Medium	17 (31%)	14 (25%)	3 (16%)
Low	28 (51%)	17 (47%)	11 (58%)
None	3 (5%)	2 (5%)	1 (5%)
Myelin loss, N (%)	55 (100%)	36 (100%)	18 (100%)

Data are n (%) for histomorphology findings

In addition to chronic de- and remyelinating pathology that was documented in the sural nerve specimens of CIDP, variable axonal loss could be found in all specimens. This axonal loss was moderate in 78% and mild in 22% of the cases as evaluated on methylene-blue stained semithin sections **(eFigure 6;** a = mild and b = moderate). Acute axonal damage was only occasionally identified (see arrowhead in eFigure 6a). In comparison, abundant acute axonal damage is shown exemplarily in a case of ANCA-associated vasculitis of the peripheral nervous system involving the sural nerve (eFigure 6c). There were no cases of end-stage axonal loss in this series and there were no noticeable differences between typical CIDP and variant CIDP specimens.

**Fig. 2:**
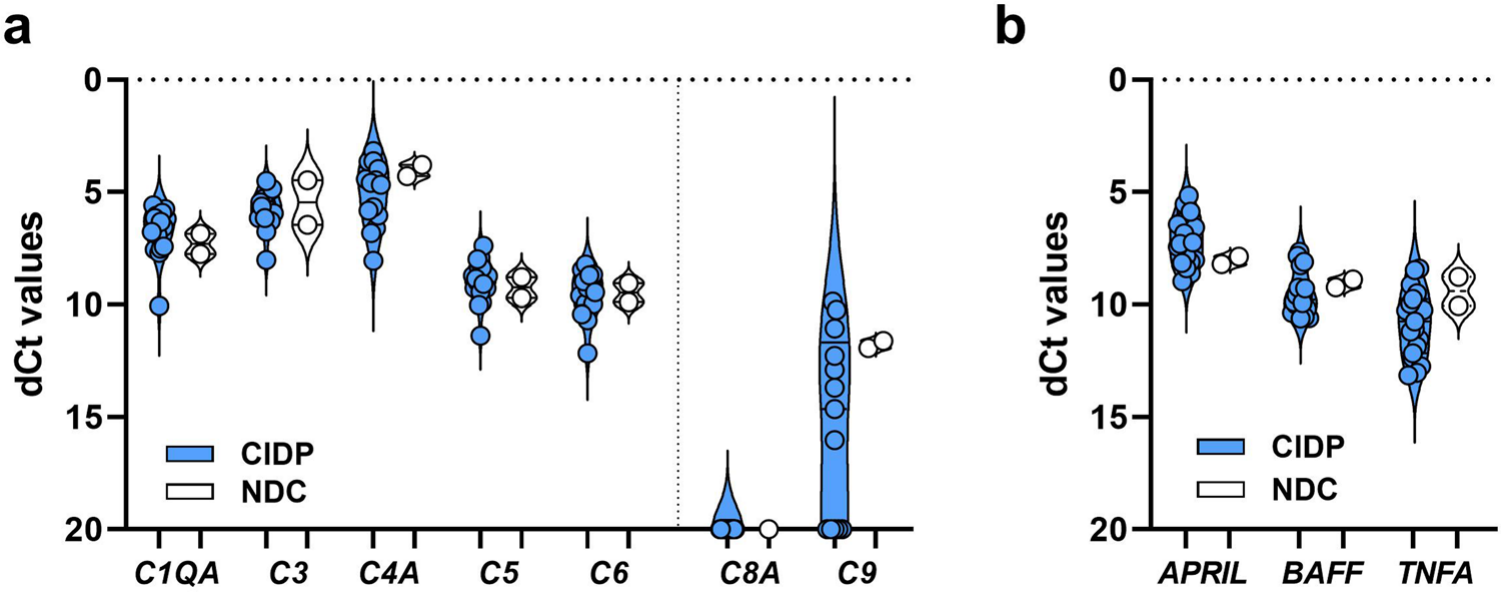
qPCR profiling of sural nerve specimens in CIDP. **a** qPCR analysis of complement activation markers of 21 CIDP and two s sural nerve tissue specimens of complement factors *C1QA*, *C4A, C5, C6, C8* and *C9*. Statistics was not performed, due to low number of controls, but *C6* seems increased in CIDP patients (~ twofold). Expression of *C9* is expressed in about 50% CIDP patients and might be increased compared to NDC. **b** qPCR analysis of the B cell markers *APRIL*, *BAFF*, as well as *TNFA* as a key factor in chronic of inflammation showed no change when compared to NDC. [7]*s:* CIDP = chronic-inflammatory demyelinating polyneuropathy, dCT = delta cycle threshold, NDC = non-diseased controls.

Sural nerve specimens from 27 patients (n = 22 with typical CIDP, n = 5 with CIDP variant) were available for RNA extraction and subsequent qPCR studies. Expression levels of complement- and inflammation-induced genes were studied to elucidate the complement-induced tissue destruction. We observed a slight up-regulation of complement factors *C6,* and in the majority of patients (64%, n = 17) an expression of *C9* **(**Fig. 2a, b**)**, the remaining 36% showed no gene expression of *C9*. There were no significant differences in expression levels of complement factors between sural nerve specimens from patients with typical CIDP and CIDP variants (see eFigure 7).

### Proteomic profiling to decipher complement factors in sural nerve specimens of CIDP patients

**Fig. 3:**
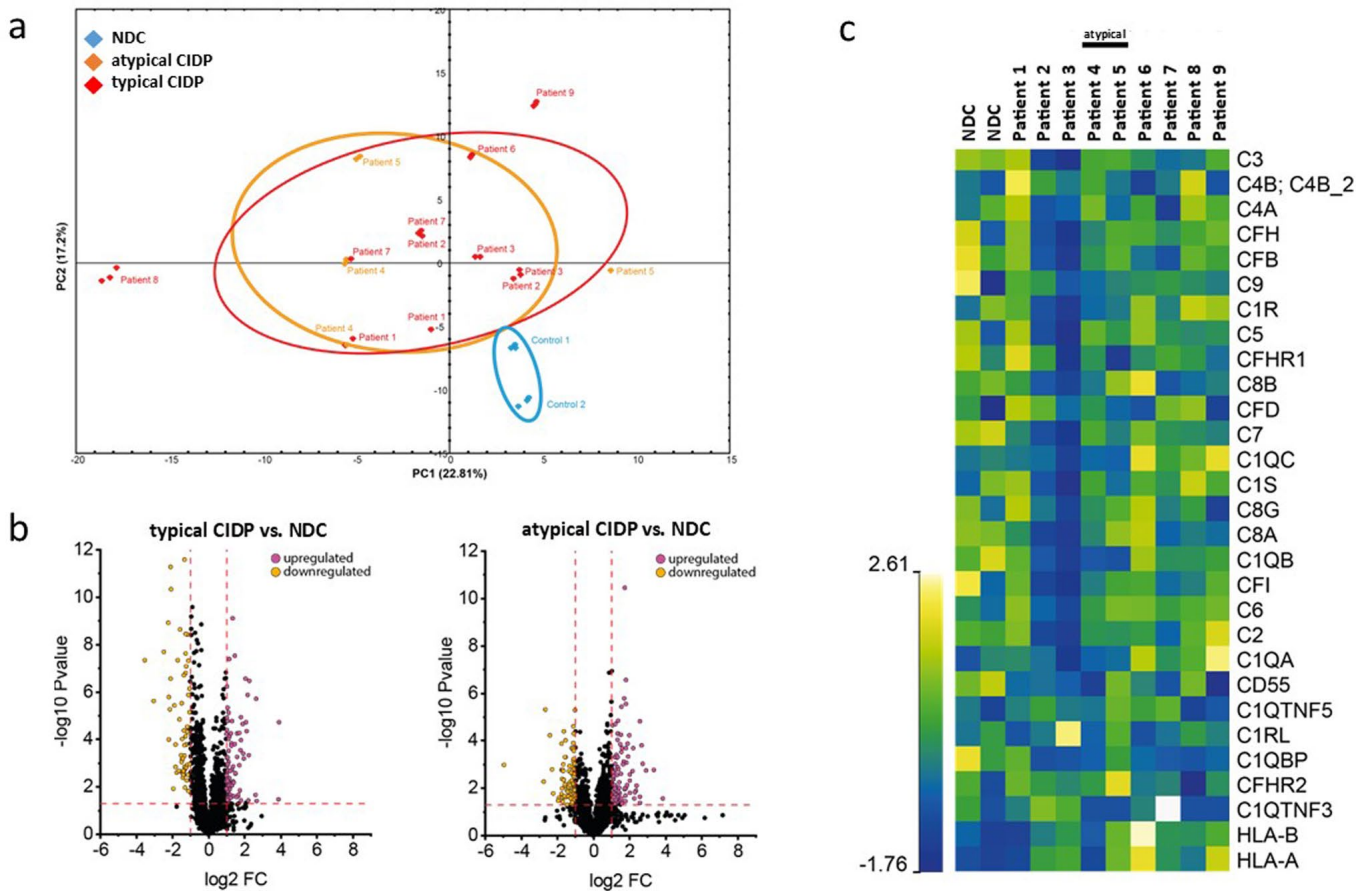
Proteomic profiling of sural nerve specimens. **a** Principal component analysis demonstrates clustering of NDCs (blue) and CIDP patients (orange = atypical CIDP; red = typical CIDP). **b** Volcano plot showing up- and downregulated proteins of typical and atypical CIDP when compared to NDCs. **c** Heat-map-based analysis of 24 proteins involved in complement pathways and HLA unveiled that all CIDP patients show an increase of C1R, C1S, C1QA, C1QC, C3, C4A, C4B, C5, C8A, C8B, C8G, C9, CFB and CFH compared to the control nerves. Patients presenting with typical (Pat. 1–3 and Pat 6–9) or atypical CIDP (Pat. 4 and 5) do not show striking differences in the complement signature or HLA proteins. *CIDP* chronic-inflammatory demyelinating polyneuropathy, *NDC* non-diseased controls.

Unbiased label-free proteomic profiling was performed exploratively on ten sural nerve specimens: seven from typical CIDP patients, two from atypical CIDP patients and two from NDC (Fig. 3a). Of the CIDP patients, five were classified having prominent complement deposition on capillaries, and four having weak. Data obtained by our data-dependent-acquisition approach filtered the quantified 1,411 proteins for factors belonging to the complement system (24 proteins) to decipher their neurodegenerative impact in CIDP further. Volcano plots of typical and atypical CIDP vs. NDC visualizes changes in abundant proteins (Fig. 3b) and the heat-map-based analysis of these 24 proteins revealed that all CIDP patients show an increase of C1R, C1S, C1QA, C1QC, C3, C4A, C4B, C5, C8A, C8B, C8G, C9, CFB and CFH compared to the abundance of these respective proteins in the control nerves (Fig. 3c). However, these protein increases vary across the nine patients.

Notably, patients #6 and #9, who had the highest complement deposition, also displayed greater clinical severity as reflected by higher INCAT scores. Other clinical characteristics, including disease subtype, age, sex or CDAS, did not differ between these groups. The two nerve biopsies derived from the patients presenting with atypical CIDP (patients #4 and #5) did not show striking differences in the complement signature or HLA proteins A and B (antigen-presenting major histocompatibility complex class I [MHCI] molecules) compared to the biopsies derived from the patients presenting with classical CIDP **(**Fig. 3**).**

### Correlation analysis of complement profiles with clinical characteristics

**Fig. 4:**
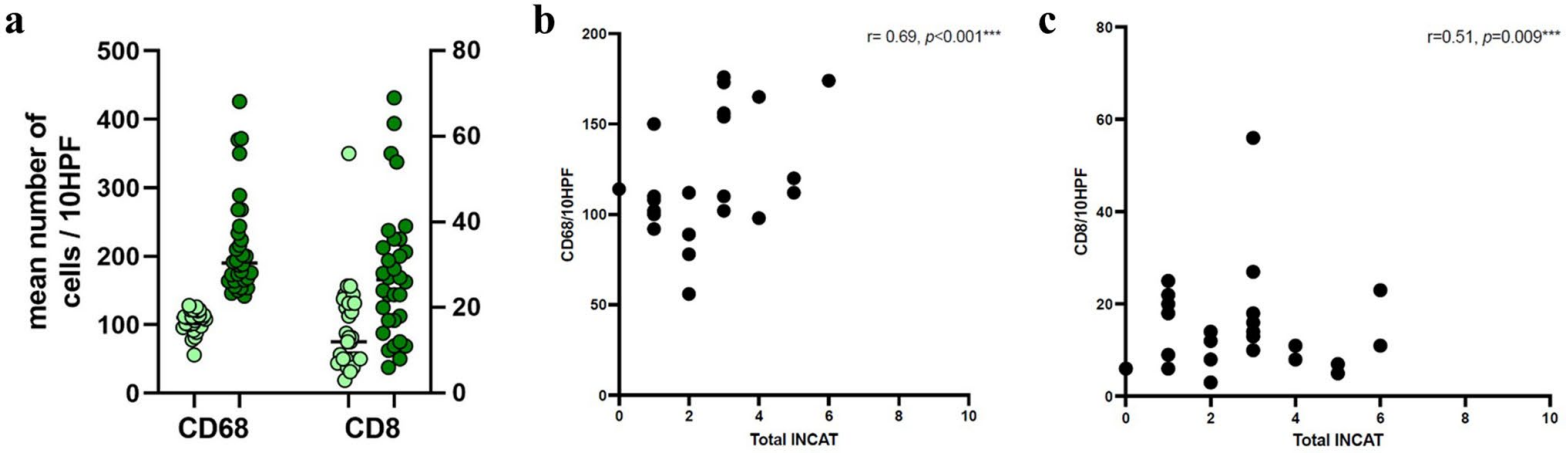
Correlation of immune cell infiltration and INCAT score. **a** Presence of endoneurial CD68 + macrophages allows division of patients into two arbitrary groups. Low numbers (up to 130 mean cells/10 HPF, light green) and high numbers (more than 130 mean cells/10 HPF, dark green). Numbers of CD8 cells are shown for the same groups (based on infiltration of CD68 + macrophages). **b** Correlation of immune cell infiltration by CD68 + macrophages and the total INCAT score. © Correlation of immune cell infiltration by CD8 + T cells and the total INCAT score

Levels of complement deposition showed no correlation with age at biopsy (r = −0.07, *p* = 0.57), sex (r = −0.19, *p* = 0.17), or disease duration (r = 0.19, *p* = 0.18). Most patients (70%, n = 44) with high to moderate complement deposition on sural nerve specimens showed a progressive disease course according to CDAS. With regard to clinical severity, there was no correlation with the INCAT-score and MRC-SS at diagnosis and 3 years after biopsy nor with treatment modality (steroids vs. IVIG). However, there was a significant positive correlation between the number of infiltrating immune cells (CD8, as well as CD68) and INCAT score (*p* < 0.001, see Fig. 4), but not with number of infiltrating CD68 + macrophages (r = −0.13, *p* = 0.378) or CD8 + T cells (r = −0.21, *p* = 0.156). CSF protein levels (r = 0.28, *p* = 0.062) and clinical symptoms (presence of sensory problems, weakness, neuropathic pain, tremor, ataxia, and cranial–nerve involvement) did not correlate with complement levels.

## Discussion

This study provides an extensive evaluation of sural nerve specimens of CIDP patients finding a marked immunohistochemical staining of C5b-9 on capillaries, regardless of the CIDP subtype, in comparison with NCD and DC. In addition, we observed a change of complement factors *C6* and *C9* by gene transcript analysis and a marked capillary de- and regeneration process as exemplified by multilayered basement membranes and fibroblast activation indicating a direct destructive process mediated by complement. Proteomic analysis identified abundance of various complement factors supporting the postulated concept of complement mediated pathways of vessel alteration.

CIDP is the most prevalent chronic autoimmune neuropathy causing substantial disability and burden of disease [37]. Due to the heterogenicity of clinical phenotypes and disease courses, there is a substantial unmet need for alternative therapies that reflect the various disease phenotypes and offer more targeted and individualized treatment options. With the advent of complement-targeted treatment options for CIDP, it is, therefore, of upmost importance to investigate the role of complement for CIDP pathogenesis.

We have found marked complement deposition primarily localized to capillary endothelial cells within the endoneurium and perineurium, with no consistent or substantial deposition observed on Schwann cells or axons. This contrasts with some previous reports describing complement accumulation on Schwann cells [28], which may in part be explained by differences in patient selection or disease stage.

Complement deposition might be a hallmark in the initiation of inflammation, as it induces fixing of immunoglobulin deposits by inducing vascular permeability changes, thereby increasing blood–nerve permeability, and enhancing access of Abs to nerve fibers [15] underscoring the above mentioned capillary pathology. We hypothesize that this vascular complement deposition may promote macrophage recruitment, which in turn could contribute to secondary demyelination of Schwann cells. Complement factor C6 features proinflammatory functions by recruiting macrophages to sites of inflammation through complement receptors and induces tissue injury through formation of the terminal complement complex C5b-9. The role of macrophage-induced demyelination is very well-characterized in CIDP [16]. Our proteomics-based quantification of complement factors did not show a consistent increase in C6 protein levels in sural nerve biopsies from our nine patients compared to the two available controls, as elevated C6 was observed in only 2 out of 9 cases. This finding may be influenced by the fact that the control samples were also collected based on clinical indications, which could be associated with subtle alterations in protein abundances. However, in line with our transcript studies, proteomics confirmed increase of C9 and, moreover, of C5 in accordance with our immunostains. Interestingly, patients with the highest overall complement deposition in the proteomic profile (patients #6 and #9) also presented with greater clinical severity, as reflected by higher INCAT scores. While this observation may suggest a link between systemic complement activation and disease burden, the small sample size and heterogeneity of the cohort preclude firm conclusions, and larger studies will be required to confirm this potential association.

Our results are further supported by preclinical data from a rat model of CIDP, where complement depletion led to reduced severity, demyelination, and inflammation [17, 47, 48]. In addition, genetic mutations of the complement regulator CD59, leading to dysfunctional MAC-dependent complement regulation, cause a CIDP-like disease in infants [32]. This might explain our proteomic findings of an up-regulation of C8B (concomitant to C8A and C8G), as the human complement regulator CD59 binds to the alpha-chain of C8 and consecutively C9 leading to MAC-dependent destruction. In addition, these patients improved upon complement inhibition further underlying the link between complement activation and acquired chronic peripheral demyelination [31].

Complement capture and inhibition is further the main mechanisms of action of IVIg, the currently most widely used first-line therapy in CIDP [13, 44]. IVIG is thought to act through several pathways, including complement inactivation and neutralization of idiotypic antibodies [12]. There is solid evidence that the anti-inflammatory activity of IVIg in CIDP crucially depends on the presence and up-regulation of FcγRIIB, which is the main driver of complement activation of the IgG fragment [27, 42, 45]. It has been further shown that untreated patients with CIDP, compared with demographically matched healthy controls, showed consistently lower FcγRIIB expression levels on naive B cells and FcγRIIB expression. As FcγRIIB can reduce complement activation by capturing immune complexes, the lower expression may contribute to the higher complement deposition on sural nerves. We can exclude potential confounding by previous therapy with IVIg or steroids, as mean time of last treatment was > 6 weeks. In another study examining newly diagnosed, treatment-naïve CIDP patients, C5a and soluble terminal complement complexes in serum and CSF correlated with disease severity as measured by INCAT-score at time of diagnosis, and were reduced significantly after treatment initiation with IVIg [36]. These results might explain why we could not observe a correlation of complement deposition levels in sural nerve specimens with clinical severity, disease duration and CSF protein levels at time of biopsy, as all our patients had undergone treatment with either IVIg and/or corticosteroids before biopsy. However, we observed that patients with high and moderate complement deposition present with a progressive disease course independent of CIDP subtype, thus complement seems to play a crucial role for disease pathogenesis and progression in all CIDP phenotypes.

Many clinical trials are currently conducted in CIDP to offer more targeted treatment options that lead to better symptom control. Apart from clinically proven efficacy of the FcRn-inhibitor efgartigimod for treatment of CIDP [3], which has been recently approved for this indication, complement inhibitory therapies are under investigation. Recently, results of the open-label phase II, proof-of-concept study investigating the efficacy of the C1s-complement inhibitor riliprubart, have been presented showing promising efficacy [38]. Riliprubart, also known as SAR445088, targets active C1s protein, a C1 complex serine protease, responsible for activating the classical complement pathway. By selectively inhibiting the C1 complex, the agent suppresses the downstream activation of complement system signaling cascades that could block key inflammatory mechanisms underlying demyelination and axonal damage in CIDP.

Across all outcomes of disability and impairment, clinically meaningful improvements were seen in riliprubart-treated participants. A phase III trial is, therefore, planned (NCT06290141) [7]. In addition, further trials investigating C1s- or C2-inhibition are currently recruiting (DNTH103 NCT06858579 [6]; empasiprubart, NCT06742190 [8]**).**

This study harbors limitations. First, patients were not treatment-naïve, which might have led to lower complement protein levels, as transient complement reduction can occur after plasmapheresis [49], corticosteroid therapy [5] and IVIg treatment [4]. However, most patients had a treatment-free interval of more than 6 weeks, hereby reducing the likely impact on systemic complement levels [1]. Second, as is inherent to real-world designs, only therapy-refractory or poor-responder patients underwent nerve biopsy to exclude alternative diagnoses, introducing a potential selection bias. All included cases fulfilled the 2021 EAN/PNS diagnostic criteria for typical or atypical CIDP, and patients classified as “possible CIDP” were not included. This selection approach, while ensuring diagnostic accuracy, may still limit the generalizability of our findings to milder or treatment-responsive cases.

Third, given the long recruitment period (2006–2023), we acknowledge that autoimmune nodopathies were not recognized as a separate entity until 2021. To minimize potential diagnostic misclassification, we retrospectively tested stored serum samples for paranodal auto-antibodies (anti-NF155, anti-CNTN1, and anti-Caspr1) in all available cases from the early study period (2006–2014) and excluded any positive cases from the analysis.

Fourth, the number of healthy controls was limited—particularly for the proteomic analyses—since obtaining sural nerve tissue from completely healthy donors is notoriously challenging, and hence, our cases were an exception to this fact. However, objective clinical exams, lab-findings, electrodiagnostic, histopathological and electron microscopical results were unremarkable.

Finally, given the exploratory nature of this study, we did not include a validation cohort from other CIDP centers. While we included patients with both typical CIDP and CIDP variants to explore potential subtype-related differences in complement expression, this heterogeneity may limit the generalizability of our conclusions. These limitations should be considered when interpreting our findings.

In summary, this study provides the to date largest quantitative and qualitative analysis of complement in sural nerve specimens of CIDP patients offering valuable insights into CIDP pathogenesis. In addition, despite the limitations, we believe that the convergence of findings from immunohistochemistry, gene expression, and proteomic profiling provides a consistent picture supporting the presence of complement involvement in CIDP and therefore support the approach of targeting the complement system as a new and promising therapeutic strategy in CIDP. Further research in larger cohorts of patients is warranted to further unravel the functional implication and role of complement for CIDP progression to optimize patient care.

## Supplementary Information

Below is the link to the electronic supplementary material.

Supplementary material 1 (JPG 89.7 kb)

Supplementary material 2 (JPG 301.0 kb)

Supplementary material 3 (JPG 297.3 kb)

Supplementary material 4 (JPG 447.4 kb)

Supplementary material 5 (JPG 383.2 kb)

Supplementary material 6 (JPG 45.2 kb)

Supplementary material 7 (JPG 157.1 kb)

Supplementary material 8 (DOCX 15.9 kb)

## Data Availability

All the data and materials supporting the conclusions of this article are included in this article. Still, individual-level data can only be released under a suitable data-sharing agreement due to informed consent restrictions. The mass spectrometry proteomics data have been deposited to the ProteomeXchange Consortium via the PRIDE partner repository with the data set identifier PXD056286 [33]. This study did not generate new unique reagents or codes.
