# Supplementary figures and images for: Complement profiling of sural nerves in chronic-inflammatory demyelinating polyneuropathy
Source: Acta Neuropathol. 2025 Sep 19;150(1):32. doi: 10.1007/s00401-025-02936-w (PMC12449320; doi:10.1007/s00401-025-02936-w)

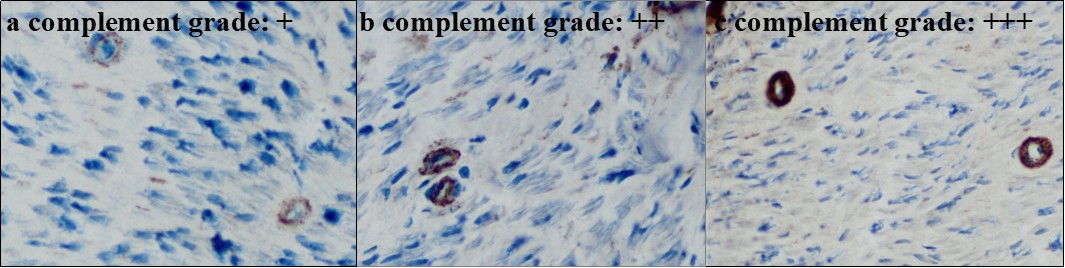

Supplement: Supplementary file 1 — Supplementary material 1 (JPG 89.7 kb) [file 401_2025_2936_MOESM1_ESM.jpg]

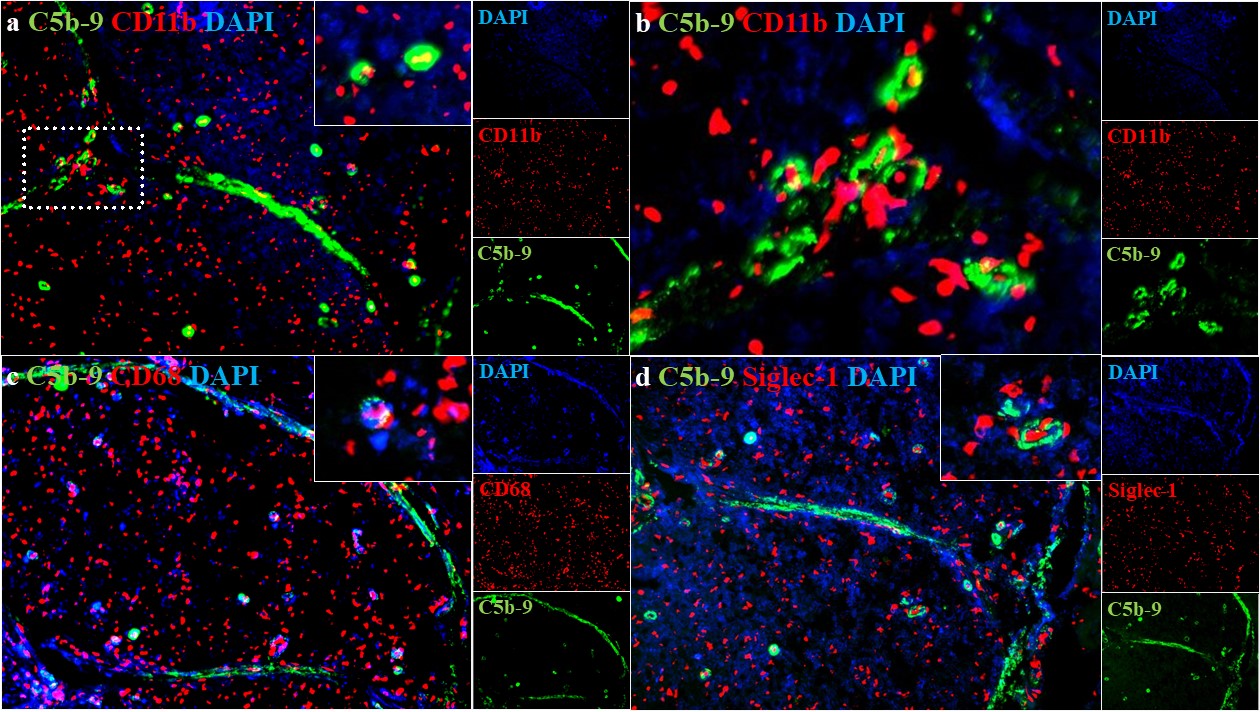

Supplement: Supplementary file 2 — Supplementary material 2 (JPG 301.0 kb) [file 401_2025_2936_MOESM2_ESM.jpg]

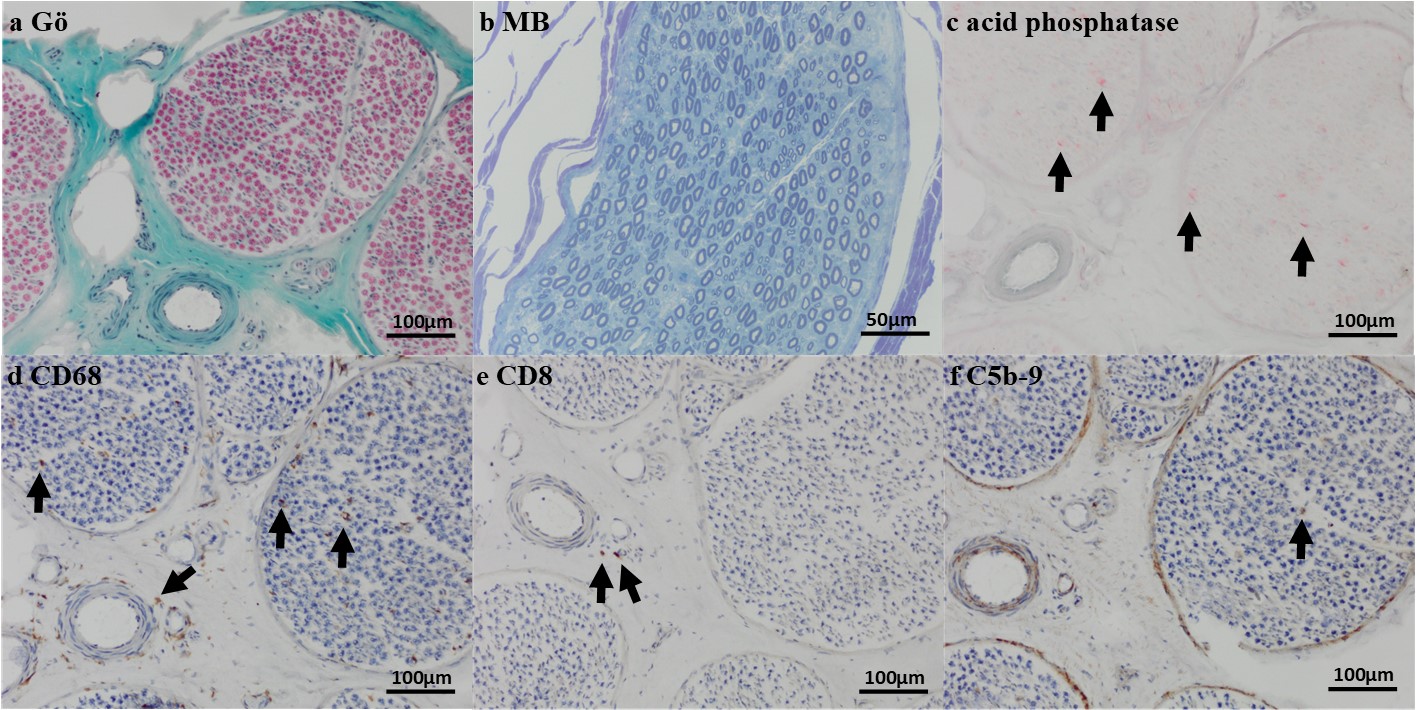

Supplement: Supplementary file 3 — Supplementary material 3 (JPG 297.3 kb) [file 401_2025_2936_MOESM3_ESM.jpg]

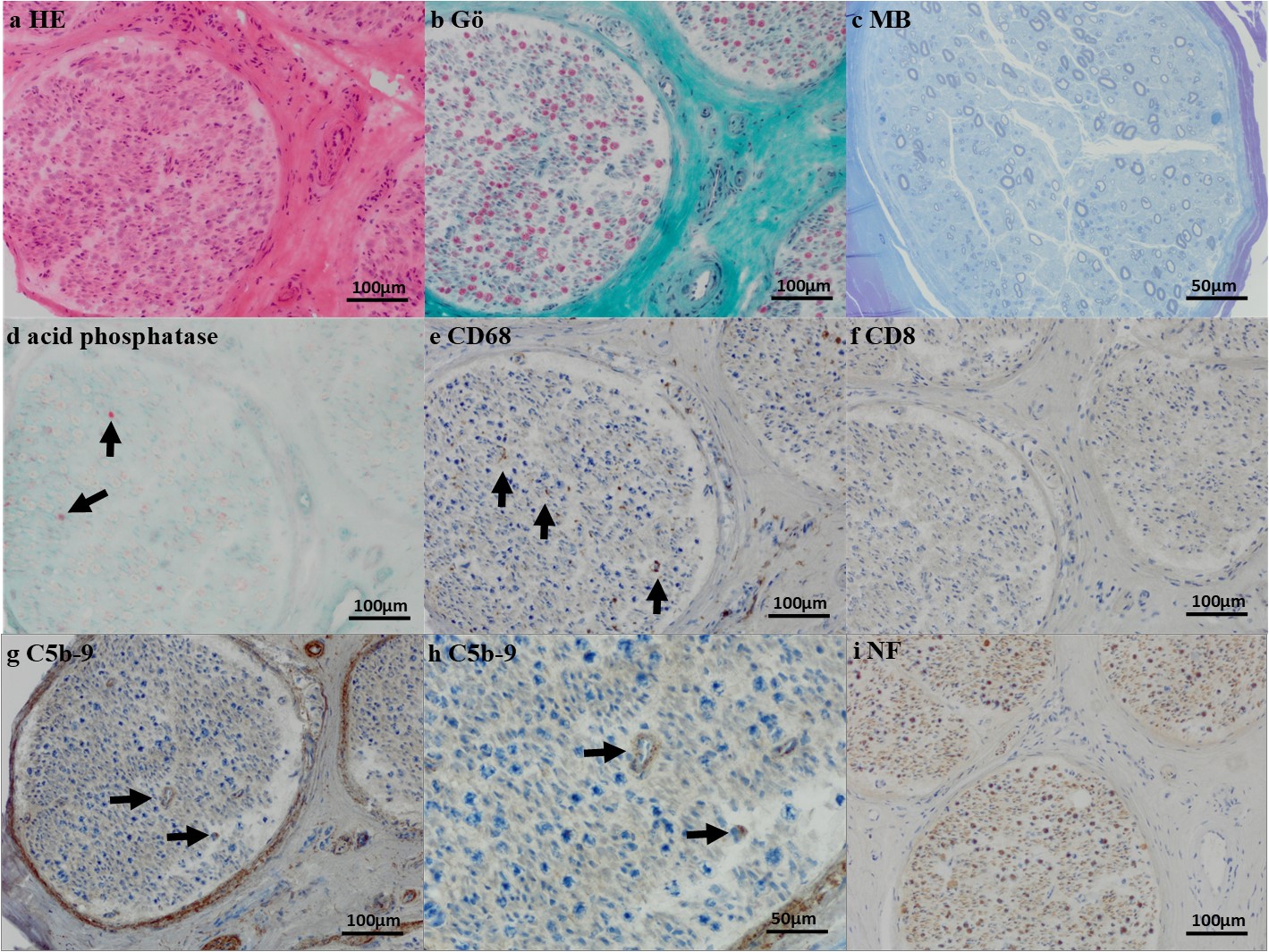

Supplement: Supplementary file 4 — Supplementary material 4 (JPG 447.4 kb) [file 401_2025_2936_MOESM4_ESM.jpg]

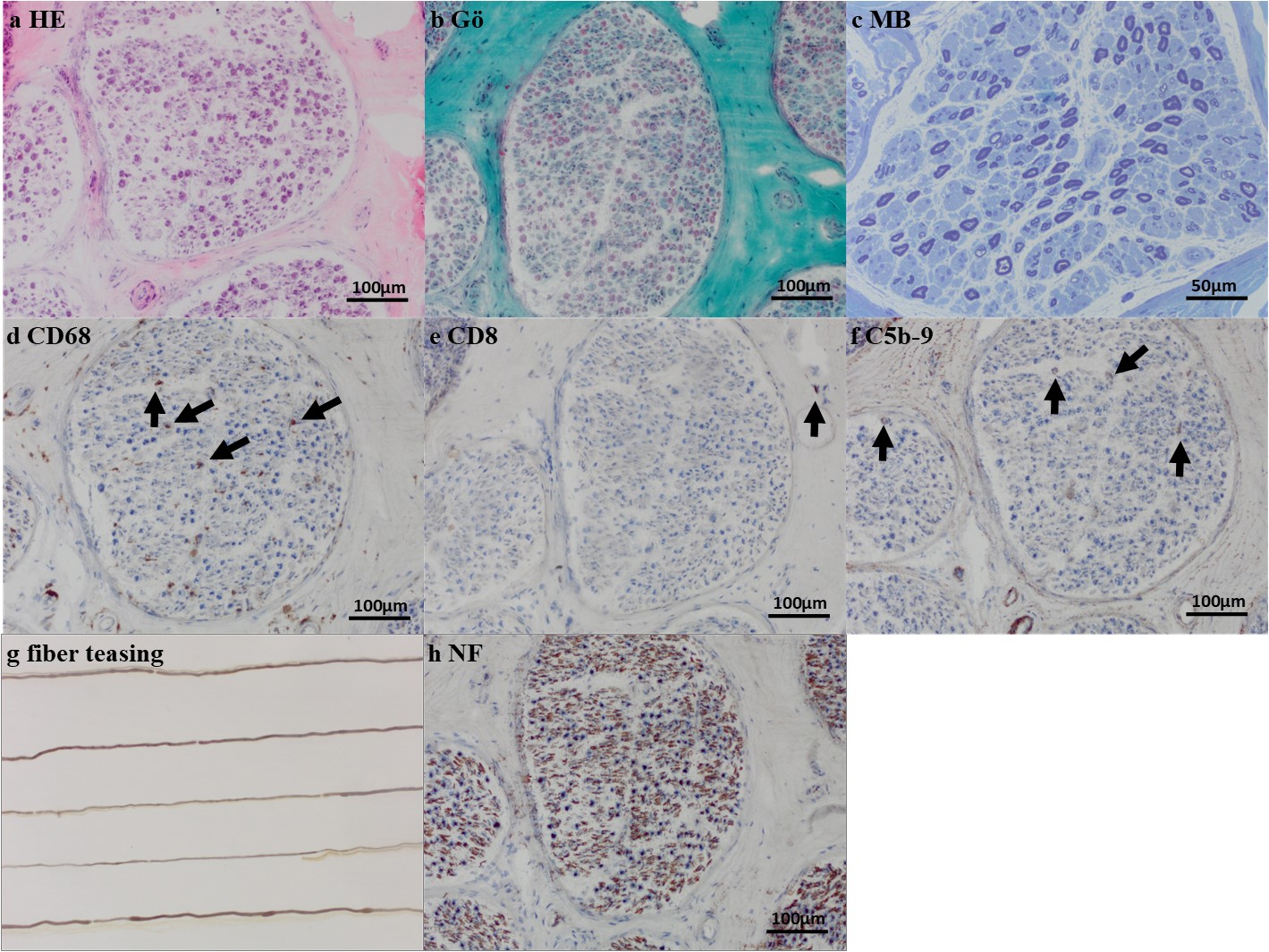

Supplement: Supplementary file 5 — Supplementary material 5 (JPG 383.2 kb) [file 401_2025_2936_MOESM5_ESM.jpg]

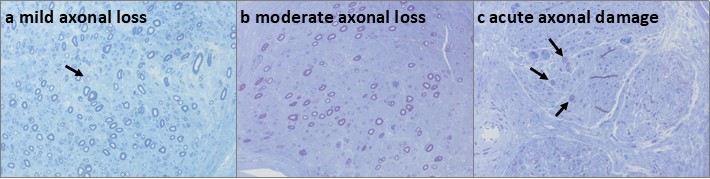

Supplement: Supplementary file 6 — Supplementary material 6 (JPG 45.2 kb) [file 401_2025_2936_MOESM6_ESM.jpg]

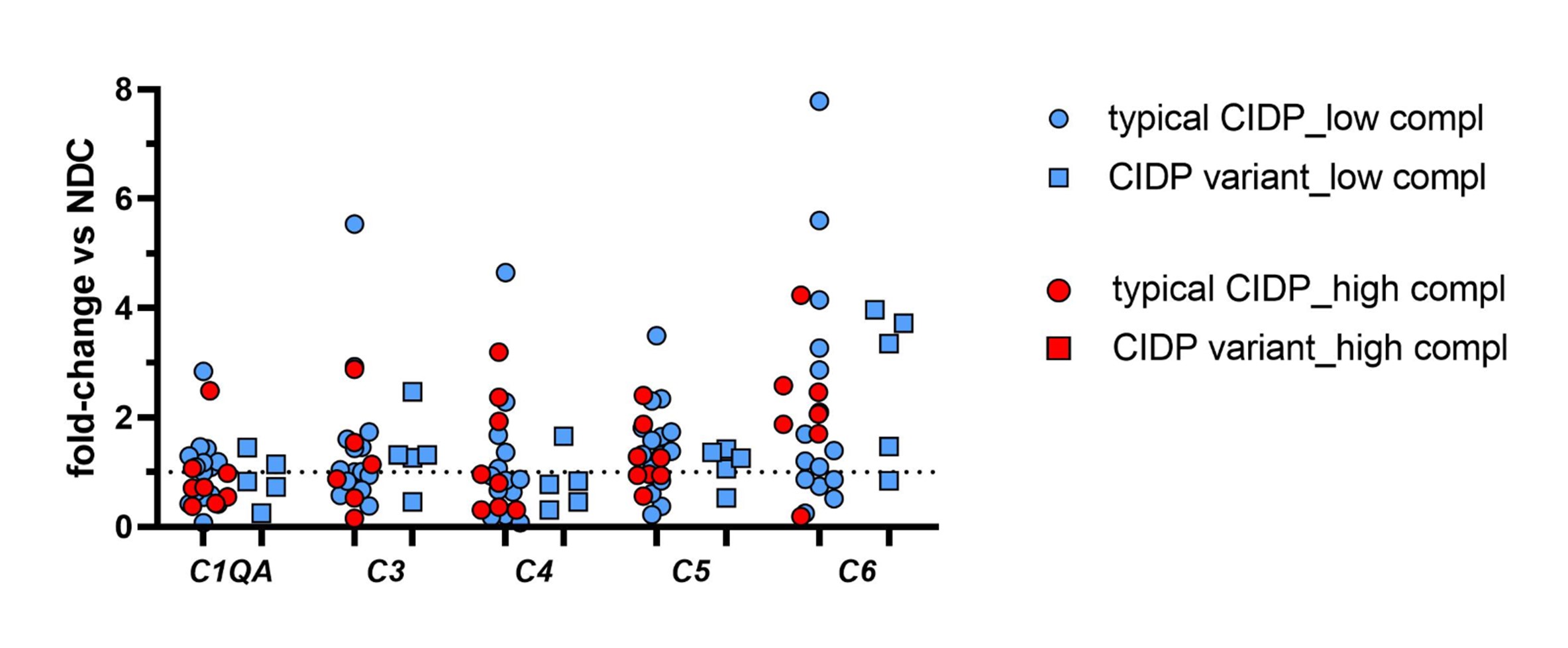

Supplement: Supplementary file 7 — Supplementary material 7 (JPG 157.1 kb) [file 401_2025_2936_MOESM7_ESM.jpg]
