# Supplementary material for: Complement profiling of sural nerves in chronic-inflammatory demyelinating polyneuropathy
Source: Acta Neuropathol. 2025 Sep 19;150(1):32. doi: 10.1007/s00401-025-02936-w (PMC12449320; doi:10.1007/s00401-025-02936-w)
Supplement: Supplementary file 8 — Supplementary material 8 (DOCX 15.9 kb) [file 401_2025_2936_MOESM8_ESM.docx]

**Supplementary material**

**eFigure 1: Semiquantitative grading of complement depositions in sural nerve biopsies**

Semiquantitative Score of C5b-9 complement deposits in sural nerve biopsies were quantified as + (low), ++ (intermediate) and +++ (high)

**eFigure 2:** **double-immunofluorescence of macrophage marker and complement in sural nerve biopsies**

The terminal complement complex C5b-9 (green) was co-stained with macrophage markers CD11b (a, b), CD68 (c) and Siglec-1 (d) in red, demonstrating proximity of macrophages around decorated capillaries.

**eFigure 3: histomorphology in control nerve**

(a) Gömöri trichrome showing nerve fascicles with a dense and regular myelination of axons, and (b) semithin sections with methylene blue stain identifying regular myelination of axons in the endoneurium and absence of any acute axonal damage or onion bulb formation or regeneration clusters. (c) only few endoneurial acid phosphatase positive (red) or (d) CD68+ macrophages are seen. (e) absence of any endoneurial CD8+ T cells, note: single T cells can appear in the epineurium, as well as (f) absence of any relevant complement staining (C5b-9) on endoneurial capillaries and

**eFigure 4: histomorphology in CMT**

(a, b) visible is demyelination, as well as axonal degeneration, as well as on (c) semithin sections with methylene blue, which shows demyelination and axonal degeneration. (d, e) few endoneurial acid phosphatase and CD68+ macrophages and (f) absence of any endoneurial CD8+ T cells are seen. (g, h) no or faintly detectable little complement staining (C5b-9) on endoneurial capillaries, while (i) neurofilament staining reveals thinned axons.

**eFigure 5: histomorphology in idiopathic neuropathy**

(a, b) idiopathic neuropathy patients demonstrate demyelination and axonal atrophy and degeneration. (c) semithin sections with methylene blue stain shows reduced density of myelinated fibers, while (d) few endoneurial CD68+ macrophages can be detected. (e) no endoneurial CD8+ T cells are seen, while single T cells might appear in the epineurium. Furthermore, (f) there is only faintly detectable complement staining (C5b-9) on endoneurial capillaries. (g) fiber teasing shows individual demyelinated nerve fibers. (h) neurofilament staining primarily highlights axonal degeneration. This presents as thinned or atrophic axons

**eFigure 6: visualization of axonal loss in CIDP and ANCA-associated vasculitis**

Visualization of axonal loss being mild (a) or moderate (b) in CIDP, as evaluated on methylene-blue stained semithin sections. Acute axonal damage was only occasionally identified (see arrowhead a). In comparison, abundant acute axonal damage is shown in a case of ANCA-associated vasculitis of the peripheral nervous system involving the sural nerve (c).

**eFigure 7: gene transcript analyses of complement factors in typical and CIDP variants**

qPCR analyzes stratified as typical CIDP, and CIDP variant patients, as well as for low vs. high complement deposits. There were no differences between subgroups on the transcriptomic level

Abbreviations: CIDP=chronic inflammatory demyelinating polyneuropathy, NDC=non-diseased controls.
